# Frequency, Timing, Risk Factors, and Outcomes of Desaturation in Infants With Acute Bronchiolitis and Initially Normal Oxygen Saturation

**DOI:** 10.1001/jamanetworkopen.2020.30905

**Published:** 2020-12-23

**Authors:** Fabiola Stollar, Alban Glangetas, Fanny Luterbacher, Alain Gervaix, Constance Barazzone-Argiroffo, Annick Galetto-Lacour

**Affiliations:** 1 General Pediatric Division, Department of Pediatrics, Gynecology and Obstetrics, University Hospitals of Geneva, Geneva, Switzerland; 2 Pediatric Emergency Division, Department of Pediatrics, Gynecology and Obstetrics, University Hospitals of Geneva, Geneva, Switzerland; 3 Pediatric Pulmonology Unit, Department of Pediatrics, Gynecology and Obstetrics, University Hospitals of Geneva, Geneva, Switzerland

## Abstract

**Importance:**

Little is known about the natural course of oxygen desaturation in acute bronchiolitis. Information on risk factors associated with desaturation as well as the time to desaturation in infants with bronchiolitis could help physicians better treat these infants before deciding whether to hospitalize them.

**Objective:**

To prospectively determine the frequency of desaturation in infants with bronchiolitis, along with the time to desaturation and risk factors associated with desaturation, and to compare infants who were hospitalized with those discharged home and evaluate risk factors for rehospitalization.

**Design, Setting, and Participants:**

This cohort study was conducted during the 2017 to 2018 and 2018 to 2019 respiratory syncytial virus seasons in a tertiary care pediatric emergency department in Switzerland. Included individuals were 239 otherwise-healthy infants aged younger than 1 year, diagnosed with acute bronchiolitis and oxygen saturation of 90% or more on arrival. Data were analyzed from July 2019 to October 2020.

**Exposures:**

After receiving triage care, study participants admitted to the emergency department were equipped with a pulse oximeter to continuously record oxygen saturation (Spo_2_ levels), regardless of subsequent hospitalization or discharge home.

**Main Outcomes and Measures:**

The primary outcome was desaturation (ie, Spo_2_ < 90%) during the first 36 hours.

**Results:**

Of 239 infants enrolled, with a median (interquartile range [IQR]) age of 3.9 (1.5-6.5) months, 116 (48.5%) were boys and desaturation occurred in 165 infants (69.0%). Median (IQR) time to desaturation was 3.6 (1.8-9.4) hours. The rate of desaturation was similar between infants hospitalized and those discharged home (137 of 200 infants [68.5%] vs 28 of 39 infants [71.8%]; difference, −3.3%; 95% CI, −18.8% to 12.2%; *P* = .85). A more severe initial clinical presentation with moderate or severe retractions was the only independent risk factor associated with desaturation (odds ratio, 2.73; 95% CI, 1.49 to 5.02; *P* = .001). Of 39 infants discharged home, 22 infants (56.4%) experienced major desaturations. However, infants with desaturations, including those with major desaturations, had rates of rehospitalization similar to those of infants without desaturations (8 of 28 infants [28.5%] vs 3 of 11 infants [27.3%]; difference, 1.2%; 95% CI, −29.9% to 32.5; *P* > .99).

**Conclusions and Relevance:**

These findings suggest that rates of desaturation in infants with acute bronchiolitis were high and similar between infants who were hospitalized and those discharged home. A more severe initial clinical presentation was the only risk factor associated with desaturation. However, for infants discharged home, desaturation was not a risk factor associated with rehospitalization.

## Key Points

**Question:**

What are the frequency, timing, risk factors, and outcomes associated with oxygen desaturation in infants with acute bronchiolitis and initially normal oxygen saturation?

**Findings:**

In this cohort study of 239 infants, desaturation occurred in most infants, regardless of whether they were hospitalized or discharged home. A more severe initial clinical presentation was the only risk factor associated with desaturation, but desaturation was not a risk factor associated with rehospitalization.

**Meaning:**

These findings suggest that desaturation in acute bronchiolitis was frequent, especially for infants with a more severe clinical presentation, but it was not a risk factor associated with rehospitalization.

## Teaser

This cohort study examines the frequency, timing, risk factors, and outcomes associated with oxygen desaturation in infants with acute bronchiolitis and initially normal oxygen saturation.

## Introduction

Although most children presenting at the emergency department (ED) with acute bronchiolitis can be treated as outpatients, the clinical course is often uncertain and there is a risk of further worsening, even among children with an apparently mild disease. This makes it difficult for physicians to determine the appropriate observation period before deciding to hospitalize patients or discharge them home.^1^ Many ED physicians rely on oximetry when deciding on hospital admission.^2,3^ However, the criteria for using oxygen therapy vary widely, with no evidence that oxygen saturation is associated with disease progression.^3,4,5,6,7,8^

Furthermore, little is known about the natural course of oxygen desaturation in bronchiolitis, and to our knowledge, there is no information on the extent to which the severity and length of desaturations can affect outcomes.^9,10^ Information on risk factors associated with desaturation in infants with bronchiolitis and on the time to desaturation could provide guidance to physicians on which course of action to follow, including hospitalization.

In a 2016 retrospective study^11^ of 581 bronchiolitis episodes in patients younger than 1 year admitted to our ED, oxygen desaturation as measured by pulse oximetry (Spo_2_ < 92%) occurred in 106 patients (18.2%). We found that female sex, age younger than 3 months, ED readmission, more severe initial clinical presentation, and higher initial Pco_2_ levels (ie, >45 mm Hg) were risk factors associated with desaturation. The median (interquartile range [IQR]) time to desaturation varied with age; patients aged younger than 3 months desaturated later than patients aged 3 months or older (6.0 [3.0-14.0] hours vs 3.0 [2.0-6.0] hours; *P* < .001).

This study aimed to prospectively determine the frequency of desaturation in infants with bronchiolitis and normal oxygen saturation on ED arrival and the time to desaturation, as well as the risk factors associated with desaturation. The secondary aims were to compare infants who were hospitalized with those discharged home and to evaluate the risk factors associated with rehospitalization.

## Methods

### Study Design

This cohort study was approved by the Human Research Ethics Committee of the Canton of Geneva. Written informed consent was obtained from all participating families. This study followed the Strengthening the Reporting of Observational Studies in Epidemiology (STROBE) reporting guideline for cohort studies. The study was conducted at the University Children’s Hospital of Geneva, a tertiary care hospital in Switzerland.

**Figure 1.**
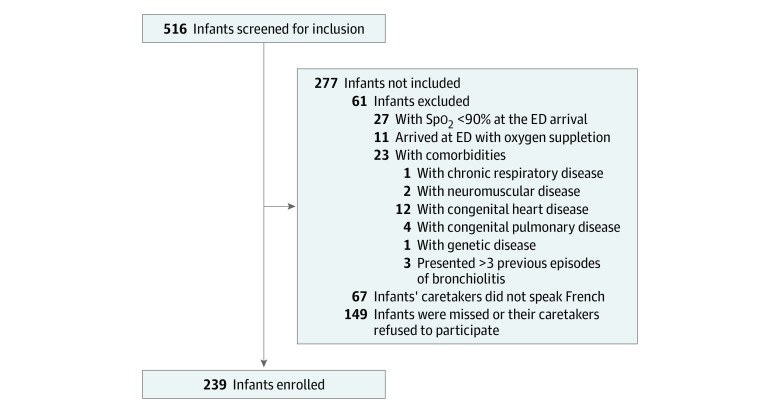
Infant Enrollment Flowchart ED indicates emergency department.

During the 2 respiratory syncytial virus (RSV) seasons of 2017 to 2018 and 2018 to 2019, infants with clinically diagnosed acute bronchiolitis were considered eligible for study inclusion if they were aged 1 year or younger and presented at the ED between 8 am and 6 pm from Monday through Friday. The diagnosis of bronchiolitis was based on a clinical presentation associated with respiratory distress, crackles, and wheezing.^12^ We excluded infants with chronic diseases, such as congenital heart disease, genetic disorders, bronchopulmonary dysplasia, congenital or acquired immunodeficiencies, and neuromuscular disorders. We also excluded infants with more than 3 previous episodes of bronchiolitis, with Spo_2_ less than 90% on ED arrival, or whose parents refused to use or were unable to understand how to use an oximeter for continuous monitoring at home. Premature infants without chronic diseases were not excluded (Figure 1).

### Data Collection and Standard of Care

Two trained residents (A. Glangetas and F.L.)were in charge of screening infants for eligibility and enrollment, as well as assigning infants for clinical findings. A structured data-collection form was used to document demographic and clinical parameters. The degree of retractions was graduated for analysis into none or mild: absence or retraction in 1 site (eg, subcostal retraction or nasal flaring) vs moderate or severe: retractions in more than 1 site (eg, supraclavicular and sternal retractions).

We used the following protocol for hospitalization criteria^12^: oral intake less than 50% of the daily nutritional amount required, Spo_2_ less than 90% in room air, progressive respiratory failure, apnea, bradycardia, and poor social conditions. Infants admitted during the study were provided with supportive care. Supplemental oxygen was administered through a nasal cannula if Spo_2_ was less than 90% in room air.

Enrolled infants were tested for RSV and influenza A and B using a Sofia fluorescent immunoassay (Quidel) as the viral diagnostic test. Capillary blood gas analysis was performed only on infants with more severe initial clinical presentation, with moderate or severe retractions, or higher respiratory rates.

The first Spo_2_ value was obtained in triage when infants arrived in the ED. Every included infant was provided with an oximeter to measure oxygen levels continuously for 36 hours after ED admission, regardless of whether they were hospitalized or discharged home. Every infant discharged home wore a portable oximeter adapted for infants (Masimo RAD-8). The RAD-8 oximeter was programmed in blind mode, so caregivers were unable to see the Spo_2_ values. Caregivers were taught how to use the oximeter at home. The oximeter automatically recorded Spo_2_ values, and our study group was responsible for retrieving the oximeters. Hospitalized infants were attached to the oximeter commonly used in our hospital (Philips IntelliVue MP5); because the Philips oximeter was connected to the nurses’ monitoring system, all desaturation events were simultaneously registered on the infant’s electronic health record. Parents generally refused the use of both oximeters, and the RAD-8 oximeter could not be used alone, because it did not connect to the nurses’ monitoring system. We performed a Bland-Altman comparison test of the 2 oximeters and found a good agreement (mean difference, 0.5; 95% CI, 0.20 to 0.81).

Oxygen saturation data were downloaded by a research assistant using Profox oximetry software version PO Masimo 2011 (Profox Associates), which was used in a 2016 research study^9^ and a 2010 research study.^13^ To ensure reliability of analyses, we also examined all desaturation recordings and performed manual artifact removal by analyzing the oximeter trace throughout the duration of the recording. Artifacts were identified by a sudden and extreme drop of saturation for a few seconds or a complete absence of recording of saturation and heart rate; these artifacts were excluded from analysis.

### Outcome Measures

The primary end point was desaturation (ie, Spo_2_ <90%) occurring within the first 36 hours after ED admission. Desaturation was defined as at least 1 documented desaturation to <90% lasting 1 minute or more. Major desaturation was defined as recurrent (ie, consisting of at least 3 desaturations to less than 90% for ≥1 minute), prolonged (ie, saturation of <90% for ≥10% of monitored time), or sustained (ie, saturation of <90% lasting ≥3 minutes continuously).^9^

The secondary end point was rehospitalization after ED discharge. A follow-up telephone call was made to caregivers at 1 week after discharge to assess whether the infant had required hospitalization for worsened symptoms of bronchiolitis within 7 days of the first ED presentation.

### Statistical Analysis

Our 2016 retrospective study^11^ found that 5 factors were associated with desaturation. In the present study, we also planned to include as many as 5 explanatory variables in the multivariate model; thus, 50 desaturation events would be the minimum number necessary to robustly measure associations between these variables and the dependent variable (ie, desaturation).

All analyses were performed using Stata statistical software version 12.0 (StataCorp). Results for categorical variables were presented as proportions with 95% CIs for differences and odds ratios (ORs). Results for continuous variables were presented as medians with IQRs.

Infants with desaturation were compared with infants without desaturation. We performed univariate analyses using *t* test, Mann-Whitney test, χ^2^ test, or Fisher exact test, as appropriate. A multivariate model included factors associated with increased risk of delayed desaturation in univariate analyses and risk factors associated with desaturation found in our retrospective study^11^ (ie, female sex, age <3 months, ED readmission, and moderate or severe retractions). We calculated interval between ED arrival time (recorded automatically by the hospital computer system) and time of desaturation. Linear regression (ie, Pearson correlation coefficient) was used to analyze correlations between tests. We analyzed correlation between duration of symptoms before ED arrival and time taken for Spo_2_ to decrease. Estimated cumulative incidence of desaturation was obtained using the Kaplan-Meier approach. All *P* values were 2-tailed, with *P* < .05 considered statistically significant. Data were analyzed from July 2019 to October 2020.

## Results

### Study Group

**Figure 2.**
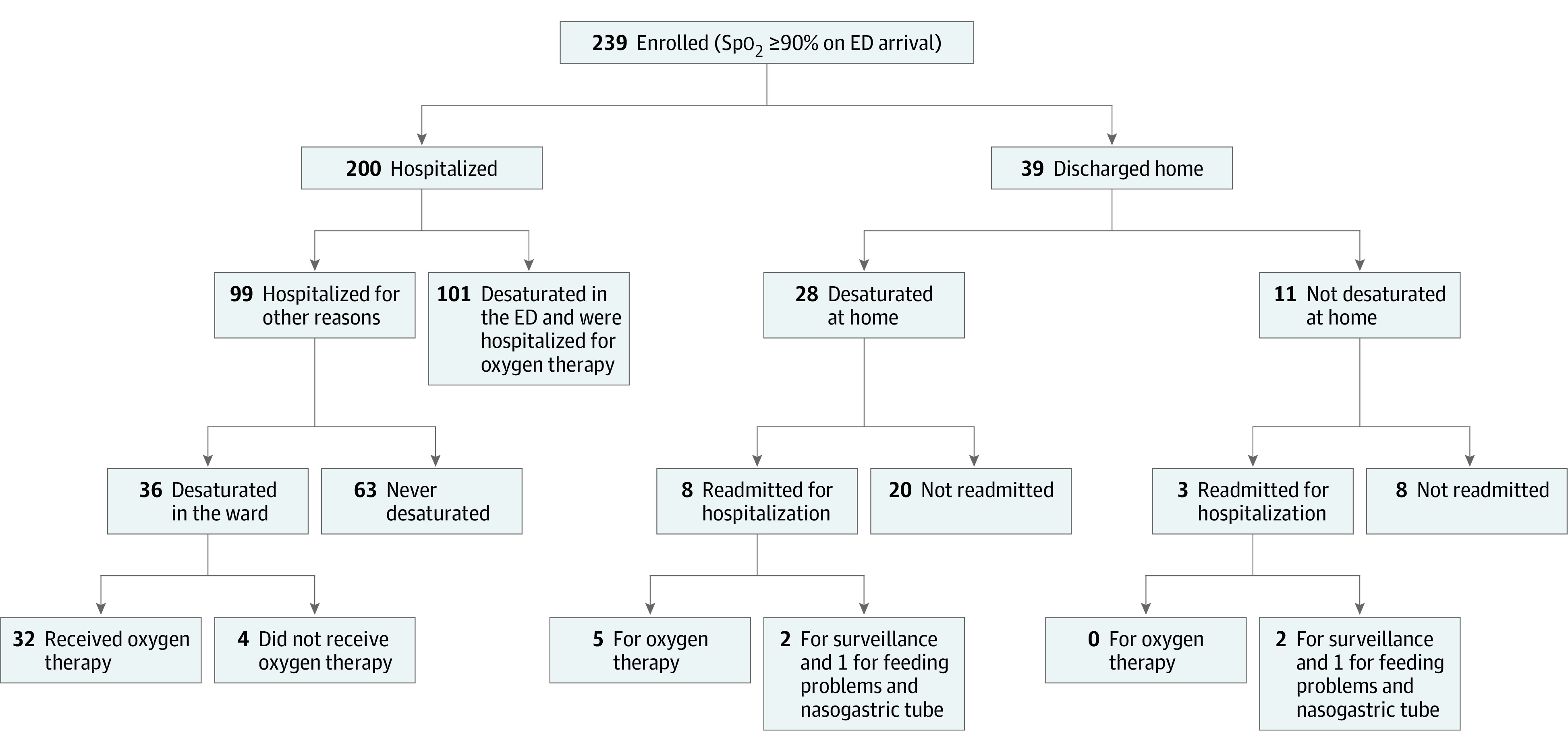
Infant Flowchart After Enrollment at Emergency Department (ED) Infants hospitalized for other reasons included 49 infants who were hospitalized for feeding problems (of whom 47 required a nasogastric tube in the ED), 9 for social reasons, and 41 for surveillance because of severe respiratory distress (of whom 24 required a nasogastric tube in the ward). Infants desaturated in the ED and hospitalized for oxygen therapy included 45 infants who also presented feeding problems (among whom 15 required a nasogastric tube in the ED and 30 required 1 in the ward). Hospitalized infants never desaturated included 1 infant who received oxygen therapy for severe respiratory distress but did not desaturate. Infants who desaturated in the ward but did not receive oxygen therapy presented desaturation as defined in our study (ie, to >90% for at least 1 minute).

During the study enrollment period, 516 infants were screened, of whom 239 infants (116 [48.5%] boys) met inclusion criteria and participated in the study (Figure 1). A further 465 infants presented outside study hours. Median (IQR) age of included infants was lower compared with infants who were not included or whose caregivers refused to participate (3.9 [1.5-6.5] months vs 5.0 [2.6-7.6] months; *P* = .002). Included infants also had higher rates of hospitalization compared with those who were not included or whose caregivers refused to participate (200 of 239 infants [83.7%] vs 83 of 204 infants [40.7%]; difference, 42.9%; 95% CI, 34.7% to 51.2%; *P* < .001). All other epidemiological characteristics were similar between groups (eTable 1 in the Supplement). After a median (IQR) observation period of 4.4 (3.2-6.2) hours, 200 infants (83.6%) were hospitalized and 39 infants (16.3%) were discharged home. Referral to the intensive care unit occurred for 19 infants who were hospitalized (9.5%). Median (IQR) hospital length of stay was 4.5 (2.6-6.9) days. Figure 2 provides a detailed flowchart of infant outcomes after enrollment at the ED. No deaths were documented.

### Frequency, Timing, Risk Factors, and Outcomes of Desaturation

**Figure 3.**
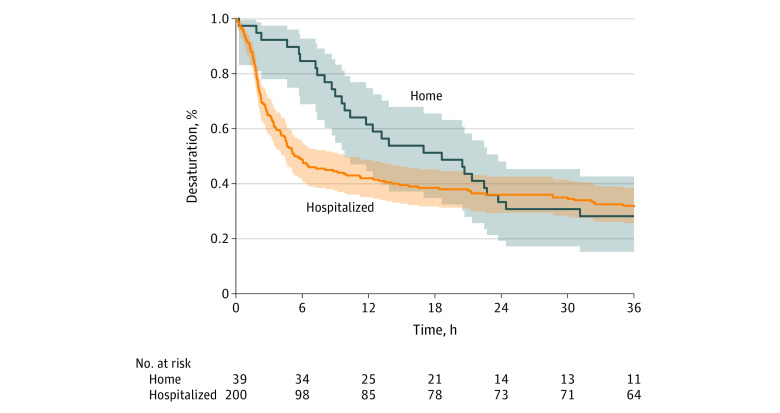
Kaplan-Meier Cumulative Incidence of Desaturation Among Infants Hospitalized vs Discharged Home

Desaturation occurred in 165 infants (69.0%), with 137 desaturations occurring in the hospital and 28 at home (Figure 2). Rate of desaturation was similar between infants hospitalized and those discharged home (137 of 200 infants [68.5%] vs 28 of 39 infants [71.8%]; difference, −3.3%; 95% CI, −18.8% to 12.2%; *P* = .85). However, hospitalized infants had a more severe initial clinical presentation, with a higher respiratory rate (113 of 200 infants [56.5%] vs 13 of 39 infants [33.3%]; difference, 23.2%; 95% CI, 6.8% to 39.5%; *P* = .008) and more moderate or severe retractions (148 of 200 infants [74.0%] vs 22 of 39 infants [56.4%]; difference, 17.6%; 95% CI, 0.8% to 34.3%; *P* = .03). Infants who were hospitalized also had earlier median (IQR) desaturation times compared with infants discharged home (2.8 [1.7-5.9] hours vs 11.0 [7.3-20.6] hours; *P* < .001) (Figure 3). Furthermore, infants who were hospitalized or discharged home with no supplemental oxygen had the same demographic and clinical characteristics (eTable 2 in the Supplement).

Median (IQR) time to desaturation was 3.6 (1.8-9.4) hours. Age was not associated with time to desaturation in infants aged under 3 months vs over 3 months (3.0 [1.7-9.1] hours vs 3.8 [2.0-9.7] hours; *P* = .33), nor was a more severe vs less severe initial clinical presentation with moderate or severe retractions at arrival (3.6 [1.9-9.1] hours vs 4.0 [1.7-11.8] hours; *P* = .71). Additionally, there was no association between duration of symptoms before ED arrival and time to desaturation (*R*^2^ = 0.0062; *P* = .32).

Table 1 compares clinical and epidemiologic characteristics of infants with and without desaturation. Based on univariate analysis and risk factors for desaturation found in our retrospective study,^11^ the following variables were included in multivariate regression: moderate or severe retractions, female sex, age younger than 3 months, and ED readmission. Although initial median (IQR) Spo_2_ was significantly lower in infants who desaturated vs those who did not (96% [94%-98%] vs 97% [95%-99%]; *P* = .002), this difference was not clinically relevant and these values were not included in multivariate regression. Because capillary blood gas analysis was performed on only 61 infants, the resulting estimate was considered unreliable and therefore not included in multivariate analysis. In multivariable regression analysis, only more severe initial clinical presentation with moderate or severe retractions was independently associated with desaturation (OR, 2.73; 95% CI, 1.49 to 5.02; *P* = .001).

**Table 1.** Characteristics and Outcomes of Infants With and Without Desaturation

Characteristic	No. (%)		Difference (95% CI)	*P* value
	Desaturation (n = 165)	No desaturation (n = 74)		
Girls	83 (50.3)	40 (54.0)	−3.7 (−0.2 to 0.1)	.67
Age, mo				
Median (IQR)	4 (1-6)	3 (1-6)	NA	.19
<3	65 (39.4)	35 (47.3)	−7.9 (−0.2 to −0.6)	.26
Premature	25 (15.1)	14 (18.9)	−3.8 (−0.1 to 0.1)	.45
Duration of symptoms at presentation, median (IQR), d	3 (2-5)	3 (2-4)	NA	.18
History of atopy	30 (18.2)	14 (18.9)	−0.7 (−0.1 to 0.1)	>.99
Family history of asthma	32 (19.4)	14 (18.9)	0.5 (−0.1 to 0.1)	>.99
RSV, No./total No. (%)	113/143 (79.0)	47/65 (72.3)	6.7 (−0.1 to 0.2)	.29
Influenza A, No./total No. tested (%)	7/135 (5.2)	7/62 (11.3)	−6.1 (−0.2 to <0.1)	.14
Influenza B, No./total No. tested (%)	7/135 (5.2)	1/62 (1.6)	3.6 (<0 to 0.1)	.44
Tobacco environment	33 (20.0)	17 (23.0)	−3.0 (−0.1 to 0.1)	.61
Reconsultation	36 (21.8)	21 (28.3)	−6.5 (−0.2 to 0.1)	.32
Respiratory rate >reference range for age^a^	93 (56.4)	33 (44.6)	11.8 (−0.2 to 0.3)	.10
Moderate or severe retractions	128 (77.5)	42 (56.8)	20.7 (0.1 to 0.3)	.001
Wheezing	63 (38.2)	27 (36.5)	1.7 (−0.1 to 0.2)	.88
Crackles	135 (81.8)	57 (77.0)	4.8 (−0.1 to 0.2)	.39
Decreased air entry	7 (4.2)	5 (6.8)	−2.6 (−0.1 to <0.1)	.52
Spo_2_ at arrival, median (IQR), %	96 (94-98)	97 (95-99)	NA	.002
Central cyanosis^b^	4 (2.4)	0	2.4 (<0.1 to 0.1)	.31
Apnea^c^	5 (3.0)	4 (5.4)	−2.4 (−0.1 to <0.1)	.46
Any ED treatment				
Inhaled albuterol	33 (20.0)	11 (14.9)	5.1 (−0.1 to 0.2)	.37
Oral corticosteroids	2 (1.2)	0	1.2 (<0 to <0.1)	>.99
Inhaled corticosteroids	0	1 (1.4)	−1.4 (<0 to <0.1)	.31
ED observation period, median (IQR), h	4.6 (3.3-6.1)	4.0 (2.8-6.8)	NA	.10
Hospitalization	137 (83.0)	63 (85.1)	−2.1 (−0.1 to 0.1)	.85
ICU	17 (10.3)	2 (2.7)	7.6 (<0.1 to 0.1)	.07
Hospital length of stay, median (IQR), d	5.0 (2.9-7.1)	3.5 (2.1-5.6)	NA	.004
Required nasogastric tube	76 (46.0)	41 (55.4)	−9.4 (−0.2 to <0.1)	.21
Pco_2_, median (IQR), mm Hg	45 (39-49)	38 (35-40)	NA	.01
Pco_2_ >45 mm Hg, No./total No. tested (%)	22/44 (50.0)	2/17 (11.8)	38.2 (0.2 to 0.6)	.008

Abbreviations: ED, emergency department; ICU, intensive care unit; IQR, interquartile range; NA, not applicable; RSV, respiratory syncytial virus; Spo_2_, oxygen saturation as measured by pulse oximetry.

^a^ Normal breathing values by age group: ages 0 to 1.9 months, 45 breaths per minute; 2 to 5.9 months, 43 breaths per minute; and 6 to 11.9 months, 40 breaths per minute.

^b^ Central cyanosis was defined as bluish discoloration around the core, lips, and tongue.

^c^ Apnea was defined as cessation of breathing for more than 20 seconds.

### Rehospitalization After Initial ED Discharge

Among 39 infants discharged home, 11 infants (28.2%) were rehospitalized. Infants with and without desaturations had comparable rates of rehospitalization (8 of 28 infants [28.5%] vs 3 of 11 infants [27.3%]; difference, 1.2%; 95% CI, −29.9% to 32.5%; *P* > .99).

Of 39 infants who were discharged home, 22 infants (56.4%) presented major desaturations. We found no difference between infants who were rehospitalized and those who were not rehospitalized in number of desaturation episodes or percentage of major desaturations. Additionally, there was no statistically significant difference between infants who were rehospitalized and those who were not rehospitalized in the presence of respiratory distress (ie, moderate or severe retractions) (Table 2). Of 11 readmitted infants, 5 infants (45.4%) were hospitalized for oxygen therapy, 2 infants (18.2%) for a nasogastric tube, and 4 infants (36.4%) for monitoring owing to respiratory distress (Figure 2).

**Table 2.** Desaturation Rates in Infants Requiring Rehospitalization vs No Rehospitalization After Being Discharged Home

Characteristic	No. (%)			Difference (95% CI)	*P* value
	Total (n = 39)	Rehospitalization (n = 11)	No rehospitalization (n = 28)		
Desaturation	28 (71.8)	8 (72.7)	18 (64.3)	8.4 (−0.2 to 0.4)	.72
Major desaturation	22 (56.4)	8 (72.7)	14 (50.0)	22.7 (−0.1 to 0.6)	.29
Sustained	9 (23.1)	2 (18.2)	7 (25.0)	−6.8 (−0.4 to 0.2)	>.99
Recurrent	10 (25.6)	4 (36.4)	6 (21.4)	15.0 (−0.2 to 0.5)	.42
Prolonged	3 (7.7)	2 (18.2)	1 (3.6)	14.6 (−0.1 to 0.4)	.19
First Spo_2_ desaturation value, median (IQR), %	87 (85-88)	86 (85-87)	88 (84-89)	NA	.33
First desaturation, median duration (IQR), min	4.0 (2.0-9.2)	8.5 (2.5-22.5)	4.0 (2.0-5.8)	NA	.27
Desaturation episodes, median No. (IQR)	4 (3-7)	6 (5-9)	3 (2-6)	NA	.07
Moderate or severe retractions	7 (18)	7 (63.6)	14 (50.0)	13.6 (−0.2 to 0.5)	.50

Abbreviations: IQR, interquartile range; NA, not applicable; Spo_2_, oxygen saturation as measured by pulse oximetry.

## Discussion

This cohort study found a higher frequency of desaturation compared with our retrospective study^11^ (165 of 239 infants [69.0%] vs 106 of 581 infants [18.2%]). We also found a high frequency of major desaturations at home (22 of 39 infants discharged home [56.4%]). Because the threshold of Spo_2_ desaturation was reduced from less than 92% in the retrospective study to less than 90% in the present study, we expected to find a lower frequency of desaturations; moreover, in our retrospective study, we did not retrieve desaturation data from infants discharged home. Surprisingly, whether hospitalized or discharged home, infants in the present study had the same frequencies of desaturation.

Our study found that median time to desaturation for all infants, independently of age, was 3.6 hours. The median time to desaturation found in this prospective study was similar to that found in our retrospective study^11^ considering all infants independently of age (median [IQR], 3.6 [1.8-9.4] hours vs 3.8 [2.2-7.8] hours; *P* = .86). However, in the present study, no differences in time to desaturation were observed between infants aged younger than 3 months and those aged 3 months or older. More severe initial clinical presentation and longer history of symptoms in the period running up to ED admission were not associated with changes in time to desaturation. In our retrospective study,^11^ a respiratory rate above reference range was a risk factor associated with faster desaturation in infants aged younger than 3 months.

As in our retrospective study,^11^ a more severe initial clinical presentation, with moderate or severe retractions, was a risk factor associated with desaturation.

In the present study, desaturation was not a risk factor associated with rehospitalization; moreover, even major desaturations were not associated with rehospitalization. Our results were very similar to those found in a study by Principi et al,^9^ which found that most infants (63.5%) with bronchiolitis experienced desaturations at home and that 52.5% presented major desaturations. Principi et al^9^ also found that children with and without desaturations had comparable rates of return for care, with no difference in unscheduled return medical visits or delayed hospitalizations. However, the authors did not assess time to desaturation, and their results could not be generalized to inpatient populations.

Currently, the decision on whether to hospitalize children with bronchiolitis is primarily influenced by desaturations measured using pulse oximetry, despite its questionable diagnostic value in defining illness severity.^14^ It is noteworthy that most infants in our study who desaturated after being discharged home did not require rehospitalization, whereas if they had been in a monitored hospital setting at the time of desaturation, they would have undergone significant medical interventions. Many studies have shown that relying on oximetry as a major determinant in decisions to hospitalize infants with bronchiolitis was associated with significantly increased costs, patient harm, and hospitalization rates.^8,15,16^

In our 2016 retrospective study,^11^ we proposed a 5-step guide, based on the risk factors associated with desaturation and time to desaturation, for safely discharging infants with bronchiolitis. Based on confirmations obtained in the present prospective study, detecting a desaturation was not as important as expected in helping physicians determine the appropriate observation period before deciding on hospitalization or discharge home. Indeed, desaturation was not a risk factor associated with rehospitalization, and infants who were hospitalized showed the same rate of desaturation as those discharged home. The decision to discharge home or to hospitalize should be based more on clinical presentation than on Spo_2_ value alone. Patients with deterioration in respiratory status should be hospitalized. However, as reported in a 2016 study^9^ and a 2015 study,^17^ infants with bronchiolitis who are deemed suitable for discharge home based on respiratory and hydration status should not undergo further oximetry. Missed desaturations are likely clinically unimportant in a satisfactory overall clinical status. Caregivers should be advised to return for a reevaluation if respiratory distress worsens or if the infant is consuming less than 50% of the required daily nourishment.

Further studies are necessary to better evaluate the validity of the threshold of an Spo_2_ of 90% for hospitalization and the consequences of desaturation at home. Additionally, there is a need to better define how pulse oximetry can be integrated into bronchiolitis management algorithms.

### Limitations

This study has some limitations. First, recruitment of infants for the discharged home group was frequently hindered by parents' incapacity or refusal to use the oximeter, and this resulted in more infants being included in the hospitalized group. Included infants were younger than in those in the not included or refused to participate group, likely also because we included more hospitalized infants. However, the advantage to this was that we were able to include more infants with a more severe initial clinical presentation of bronchiolitis. Indeed, for this population, there is a lack of guidance on whether to hospitalize or discharge home. Second, the oximeter (RAD-8) used in infants discharged home could not be added to the oximeter used in the hospital, because most parents refused to have their child attached to both oximeters during hospitalization; additionally, the RAD-8 could not be used alone, because it did not connect to the ward’s monitoring system. The use of different oximeters in the hospital vs at home may have led to misclassification bias. However, the test of similarity between the 2 measures, performed in the hospital setting, suggested that this bias was low and nondifferential, with the same proportion of infants being wrongly classified as desaturated as the proportion of infants being wrongly classified with an appropriate level of saturation. Thus, the difference in groups may be slightly underestimated.

## Conclusions

This cohort study found that a significant proportion of infants with acute bronchiolitis admitted to the ED exhibited oxygen desaturation, regardless of whether they were hospitalized or discharged home. The median time to desaturation was 3.6 hours, confirming results from our 2016 retrospective study. The only risk factor associated with desaturation was a more severe initial clinical presentation with moderate or severe retractions. Desaturation was not a risk factor associated with rehospitalization.

## Notes

Supplement. **eTable 1.** Demographic and Clinical Characteristics and Outcomes of Infants Included vs Not Included or Refused to Participate in the Study **eTable 2.** Demographic and Clinical Characteristics of Infants Not Requiring Oxygen Therapy Stratified by Hospitalized vs Not Hospitalized
