# Supplementary material for: Frequency, Timing, Risk Factors, and Outcomes of Desaturation in Infants With Acute Bronchiolitis and Initially Normal Oxygen Saturation
Source: JAMA Netw Open. 2020 Dec 23;3(12):e2030905. doi: 10.1001/jamanetworkopen.2020.30905 (PMC7758807; doi:10.1001/jamanetworkopen.2020.30905)
Supplement: Supplement. — eTable 1. Demographic and Clinical Characteristics and Outcomes of Infants Included vs Not Included or Refused to Participate in the Study eTable 2. Demographic and Clinical Characteristics of Infants Not Requiring Oxygen Therapy Stratified by Hospitalized vs Not Hospitalized [file jamanetwopen-e2030905-s001.pdf]

Supplemental Online Content

Stollar F, Glangetas A, Luterbacher F, Gervaix A, Barazzone-Argiroffo C, Galetto-Lacour A. Frequency, timing, risk factors, and outcomes of desaturation in infants with acute bronchiolitis and initially normal oxygen saturation. *JAMA Netw Open*. 2020;3(12):e2030905. doi:10.1001/jamanetworkopen.2020.30905

eTable 1. Demographic and Clinical Characteristics and Outcomes of Infants Included vs Not Included or Refused to Participate in the Study

eTable 2. Demographic and Clinical Characteristics of Infants Not Requiring Oxygen Therapy Stratified by Hospitalized vs Not Hospitalized

This supplemental material has been provided by the authors to give readers additional information about their work.

© 2020 Stollar F et al. *JAMA Network Open.*

eTable 1. Demographic and Clinical Characteristics and Outcomes of Infants Included vs Not included or Refused to Participate in the Study

	Included (n=239)	Missed /Refusal/Did not speak French (n=204)[a]	Difference (95% CI)	P value
Female gender, No. (%)	123 (51.5)	86 (42.2)	9.3 (0.0003 to 0.19)	0.06
Age, median (IQR), months	3.9 (1.5-6.5)	5.0 (2.6-7.6)	-	0.002
Premature, No. (%)	39 (16.3)	23 (11.3)	5.1 (-0.01 to 0.11)	0.13
Duration of symptoms at presentation, median (IQR), days	3 (2-5)	3 (2-5)	-	0.35
Hospitalization, No. (%)	200 (83.7)	83 (40.7)	43 (0.35 to 0.51)	< 0.001

Abbreviations: IQR, interquartile range; CI, confidence interval

[a] 12 additional patients refused access to their medical information for research purposes and were not included in these analyses.

© 2020 Stollar F et al. *JAMA Network Open.*

eTable 2. Demographic and Clinical Characteristics of Infants Not Requiring Oxygen Therapy Stratified by Hospitalized vs Not Hospitalized

	Hospitalized, Not Requiring O_2 (n = 66)	Not Hospitalized, Not Requiring O_2 (n=34)	Difference (95% CI)	P value
Female gender, No. (%)	36 (54.6)	20 (58.8)	-4.2 (-0.25 to 0.16)	0.83
Age, median (IQR), months	2 (1-6)	4 (2-5)	_	0.07
Premature, No. (%)	12 (18.1)	4 (11.8)	6.3 (-0.8 to 0.21)	0.57
Duration of symptoms at presentation, median (IQR), days	3 (2-4)	4 (3-5)	_	0.11
RSV, No. (%)	49/66 (74.2)	6/8 (75.0)	-0.8 (-0.32 to 0.31)	> 0.99
Influenza A, No. (%)	7/62 (11.3)	0/5 (0)	11.3 (0.03 to 0.19)	> 0.99
Influenza B, No. (%)	1/62 (1.6)	1/5 (20.0)	-18.4 (-0.53 to 0.17)	0.14
Reconsultation, No. (%)	17 (25.8)	8 (23.5)	2.3 (-0.15 to 0.20)	> 0.99
Respiratory rate higher than normal for age, No. (%) [a]	34 ((51.5)	10 (29.4)	22.1 (0.03 to 0.42)	0.05
Retractions moderate/severe, No. (%)	41 (62.1)	18 (52.9)	9.2 (-0.11 to 0.30)	0.39
Wheezing, No. (%)	24 (36.4)	18 (52.9)	-16.5 (-0.37 to 0.04)	0.13
Crackles, No. (%)	52 (78.8)	28 (82.4)	-3.6 (-0.20 to 0.13)	0.79
Decreased air entry, No. (%)	4 (6.1)	1 (2.9)	3.2 (-0.05 to 0.11)	0.66
SpO_2 at arrival, median (IQR), %	97 (96-99)	97 (95-98)	_	0.45
Central cyanosis, No. (%) [b]	0 (0)	0 (0)	_	_
Apnea, No. (%) [c]	4 (6.0)	0 (0)	6.0 (0.003 to 0.12)	0.30
Any ED treatment, No. (%)				
Inhaled albuterol	8 (12.1)	8 (23.5)	-11.4 (-0.28 to 0.05)	0.16
Oral corticosteroids	0 (0)	0 (0)	_	_
Inhaled corticosteroids	1 (1.5)	0 (0)	1.5 (-0.1 to 0.04)	> 0.99
ED observation period, median (IQR), hours	3.9 (2.8-6.9)	4.0 (2.8-6.4)	_	0.79

Abbreviations: RSV, respiratory syncytial virus; IQR, interquartile range; CI, confidence interval; ED, emergency department

[a] Normal breathing values by age group: 0 to 1.9 months of age, 45 breaths per minute; 2 to 5.9 months of age, 43 breaths per minute; 6 to 11.9 months of age, 40 breaths per minute

[b] Central cyanosis was defined as bluish discoloration around the core, lips, and tongue

[c] Apnea was defined as cessation of breathing for more than 20 seconds

© 2020 Stollar F et al. *JAMA Network Open.*
